# Current uses and knowledge of medicinal plants in the Autonomous Community of Madrid (Spain): a descriptive cross-sectional study

**DOI:** 10.1186/s12906-020-03089-x

**Published:** 2020-10-14

**Authors:** Marta Sánchez, Elena González-Burgos, Irene Iglesias, Rafael Lozano, María Pilar Gómez-Serranillos

**Affiliations:** 1 Department of Pharmacology, Pharmacognosy and Botany, Faculty of Pharmacy, Universidad Complutense de Madrid (UCM), Madrid, Spain; 2 Department of Chemistry in Pharmaceutical Sciences, Faculty of Pharmacy, UCM, Madrid, Spain

**Keywords:** Medicinal plants, Autonomous Community of Madrid (CAM), Spain, Consumption patterns

## Abstract

**Background:**

The usage of medicinal plants as a key component of complementary and alternative medicine, has acquired renewed interest in developed countries. The current situation of medicinal plants in Spain is very limited. This paper provides new insights and greater knowledge about current trends and consumption patterns of medicinal plants in the Autonomous Community of Madrid (Spain) for health benefits.

**Methods:**

A descriptive cross-sectional study was designed for a population-based survey on medicinal plants. The data were collected (May 2018 to May 2019) using semi-structured face-to-face interviews in independent pharmacies, hospital centers and primary care health centers in the Autonomous Community of Madrid. The survey had 18 multiple choice and open-ended questions. Quantitative indices were calculated: Fidelity Level (FL), Use Value (UV) and Informants Consensus Factor (ICF). Chi-square test was used for data analysis.

**Results:**

Five hundred forty-three people were interviewed. The majority of the participants (89.6%) have used medicinal plants to treat health disorders in the past 12 months, mainly for digestive problems, sleep disorders and central nervous system diseases. A total of 78 plants were recorded, being *Matricaria recutita*, *Valeriana officinalis*, *Tilia* spp. and *Aloe vera* the most used. The highest UV was found for *Mentha pulegium* (UV 0.130) followed by *Aloe vera* (UV 0.097) and *Vaccinium macrocarpon*. (UV 0.080). The highest FL values were for *Eucalyptus* spp. (FL 90.47%) for respiratory conditions and, *Matricaria recutita* (85.55%) and *Mentha pulegium* (84.09%) for digestive problems. The highest ICF corresponded to metabolism and depression (ICF = 1), pain (ICF = 0.97), insomnia (ICF = 0.96) and anxiety (ICF = 0.95). Participants mostly acquired herbal medicines from pharmacies, herbal shops and supermarkets. Some side effects (tachycardia, dizziness and gastrointestinal symptoms) and potential interactions medicinal plants-drugs (*V. officinalis* and benzodiazepines) were reported.

**Conclusion:**

Many inhabitants of the Autonomous Community of Madrid currently use herbal products to treat minor health problems. The most common consumer pattern are young women between 18 and 44 years of age with higher education. In order to confirm the pattern, further research should be focused to investigate current uses of medicinal plants in other Spanish regions.

## Background

Complementary and alternative medicines (CAMs) represent different resources that complement or replace conventional therapies [1]. The World Health Organization’s (WHO) strategy, 2014–2023, aims to strengthen the role of traditional medicine, emphasizing the importance of promoting and including the utilization of medicinal plants in the health systems of its member countries [2].

The use of medicinal plants has acquired a renewed interest in developed countries and constitutes the first therapeutic strategy for 80% of developing countries. The majority of the global population (87.5%) uses traditional herbal medicine to treat health difficulties [3, 4]. Moreover, the growing interest in the employ of medicinal plants is evidenced by the increase of systematic reviews and prevalence surveys about herbal medicines in the last 15 years [5]. In Europe and throughout the Mediterranean area, both wild-collected and purchased from herbalists, supermarkets and pharmacies, is re-emerging. This renewed interest in traditional herbal medicine in more developed societies must be seen in the context of changes in the lifestyle, in which it enhances the concept of real and natural products. This leads consumers to perceive herbal medicine as a softer option for health issues [5–7].

Previous studies on medicinal plants in Spain are alternatively based on their traditional use [6, 7]. All these preceding works aim to study the relationships between plants and human beings in the present and in the past, based on the understanding of herbal remedies which were traditionally used to treat disorders in different health situations [8]. However, the available information on current perspectives and uses of medicinal plants in Spain is very limited compared to other European countries and USA [9, 10] and additionally very restrictive to specific areas [11, 12].

On the other hand, there exists a widespread belief among population that herbal products, being from natural origin, are not harmful to health [13]. However, medicinal plants can interact with other drugs and thus cause adverse reactions [13, 14]. The complete monographs of the German Commission E: *Therapeutic Guide to Herbal Medicines* includes more than 100 plants historically employed for their therapeutic properties but they are no longer recommended, since scientific evidence has shown potential toxicity or inefficiency [15].

Therefore, based on the state of the art, the aim of this study is to comprehend and deepen the current uses (consumption patterns, perceptions and attitudes) of medicinal plants in different regions of the Autonomous Community of Madrid (Spain), identifying the risks and precautions associated with its use and/or concomitant with conventional drugs.

## Methods

### Study area

The Autonomous Community of Madrid is the most densely populated territory in Spain (676 inhabitants per km^2^), it hosts the capital of Spain (Madrid). Most of the population is concentrated in Madrid Capital City and in its surrounding metropolitan areas. Even rural areas have Madrid as their referent in the urban lifestyle. The Autonomous Community of Madrid has a very diverse population in terms of its origin (being most of it from other Autonomous Communities), its cultural and socioeconomic terms [16]. This study has tried to represent different random localities with different social environments. In order to determine if the sample surveyed was representative of the population, the latest statistical data available on the website of Institute of Social Sciences (http://www.madrid.org/iestadis/) related to sex, age and occupation were analyzed.

### Study setting

A descriptive cross-sectional study was designed for a population-based survey on medicinal plants. This research (PR016/04) was approved on November 2016 by the Ethics and Animal Experimentation Committee, Faculty of Pharmacy, University Complutense of Madrid (Spain).

### Questionnaire

The questionnaire (Additional file 1), developed in Spanish language and designed for this study, was based on previous works on medicinal plants [9, 17, 18] and reviewed by experts in traditional plant-based medicines and pharmacognosy and agreed with experts in public health in order to evaluate the structure, relevancy and clarity of the questions. Before gathering research data, a pilot study was conducted on a sample of 50 people to validate the degree of acceptance and understanding of the questionnaire. Minor modifications, based on the pilot survey, were made in the questionnaire. The final version of the questionnaire consisted on five differentiated parts with a total of 18 multiple choice and open-ended questions to achieve a better understanding of the knowledge and use of medicinal plants for health-seeking behavior. The first part with five questions collected information on demographic data, including age, gender, educational level, area of residence and occupation. The second part, with four issues, focused on the utilization of herbs for medicinal or health purposes (disease categories, frequency, therapeutic uses, types of medicinal plants – excluding multi-herbal drug combinations - and forms of administration). This part of the questionnaire included a definition of medicinal plants: “*Plants that contain properties or compounds that can be used for therapeutic purposes or those that synthetize metabolites to produce useful drugs*” [19] and, being respondents allowed freely to comment which medicinal plants they use to prevent or treat pathologies (open list of medicinal plants). Moreover, regarding the frequency of consumption, it has been considered frequent when the interviewee consumes medicinal plants at least once a month. The third part had three questions about where the consumer acquired the medicinal plants and information on their therapeutic uses. The fourth and the fifth sections containing both 3 questions, were related to the knowledge of potential side effects and identification of concomitant consumption of medicinal plants with conventional medicines, respectively.

### Data collection and sample size

**Fig. 1:**
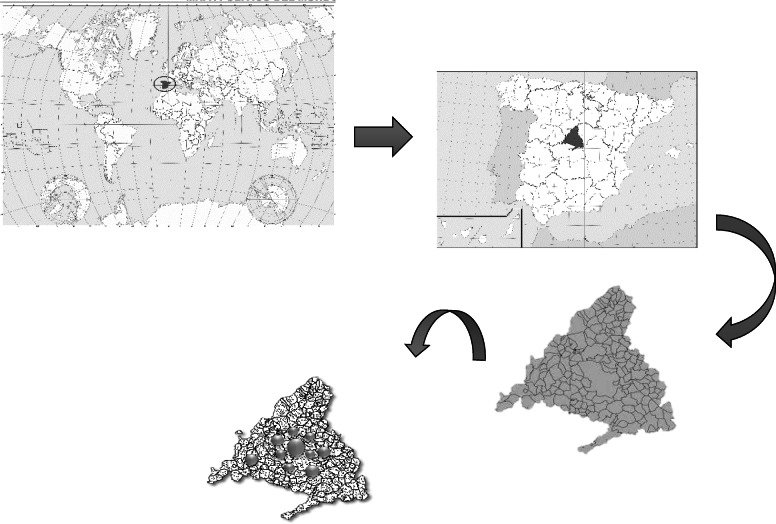
**a** Location of the Autonomous Community of Madrid in Spain. **b** Location of the municipalities within the Community of Madrid where the surveys were carried out

Data were collected on a Tablet computer by a research group from May 2018 to May 2019 using a face-to-face interview technique. Participants were recruited directly in a total of 30 independent pharmacies, hospitals and primary care health centers of different districts of the Capital City of Madrid and municipalities of the Autonomous Community of Madrid. The average number of interviewees from each place was from 15 to 20. Sample population interviewed was voluntary, randomly selected and previously informed (Fig. 1). Over the period of data collection, we conducted a total of 543 surveys. This sample size, based on population size, provides a margin error of 4% at 95% confidence level [20–22].

### Quantitative indices

The quantitative indices Fidelity Level (FL), Use Value (UV) and Informants Consensus Factor (ICF) were calculated.

#### Fidelity Level (FL)

FL corresponds to the percentage of informants that use a certain medicinal plant to treat a specific condition and it is calculated as FL (%) = (Np/N) × 100 (Np: number of informants citing a certain medicinal plant to treat a specific condition and N: number of informants citing a medicinal plant to treat any given disease) [23]. This index is used to identify the most frequently used plants to treat a disease or condition.

#### Use Value of species (UV)

UV measures the relative importance of a medicinal plant to the informants and it is calculated as UV = ƩUi/N (Ui: number of citations for each medicinal plant and N: total number of informants). It is a quantitative parameter that indicates the relative importance of the different plant species in a community. It is useful to determine plants with the greatest use (most frequently used) in the treatment of a condition. It also allows knowing the confidence in the use and pharmacological characteristics of related plants [17, 24].

#### Informants Consensus Factor (ICF)

ICF estimates the user variability of medicinal plants and it is calculated as (N_ur_ − N_t_)/(N_ur_ − 1) (N_ur_: number of used citations in each ailment category, and Nt: number of medicinal plants reported in each ailment category). This index is used to indicate to what extent the information is homogenous. The ranges obtained for this factor vary between 0 and 1. A value close to 1 indicates a relatively high use of the medicinal plant, while a low value close to 0 shows that this plant species is not used by informants for the treatment of an ICF condition. This factor was originally developed by Trotter and Logan (1986) [25] and then readapted by Heinrich et al. 1998, 2000 [26, 27].

### Data analysis

All data were entered and stored in an Excel Spreadsheet. Frequencies and percentages were calculated using Microsoft Excel. Statistical analysis was performed using chi-square tests in Sigmaplot version 14.0, to analyze data with correlations between the frequency of medicinal plants and certain demographic characteristics. The level of statistical significance was *p* < 0.05.

## Results

### Demographic information

Socio-demographic characteristics are shown in Table 1. Most participants were women (*n* = 382; 70.3%). The most frequent age from the respondents was 18–44 years (*n* = 340; 62.6%), followed by 45–64 years old (*n* = 151; 27.8%) and finally by those over 65 years (*n* = 52; 9.6%). Regarding the level of education, the majority of interviewees had higher education (*n* = 417; 76.8%) while 3.2% of participants had basic education or vocational training (i.e. auto repair, plumbing). In reference to the occupation, more than half of participants were employees (*n* = 293; 54%), followed by students (*n* = 167; 30.8%), pensioners (*n* = 56; 10.3%) and unemployed (*n* = 15; 2.8%).

**Table 1:** Demographic characteristics of participation sample

Population characteristics	Answers (***N*** = 543) n (%)
Gender	
Male	161 (29.7%)
Female	382 (70.3%)
Age (years)	
18–44	340 (62.6%)
45–64	151 (27.8%)
≥ 65	52 (9.6%)
Educational level	
Basic education	56 (10.3%)
Vocational training	70 (12.9%)
Higher education	417 (76.8%)
Occupation	
Student	167 (30.8%)
Employee	293 (54%)
Unemployed	15 (2.8%)
Pensioner	56 (10.3%)
Does not answer	12 (2.2%)

### Uses and consumption patterns of medicinal plants

The majority of the population interviewed (*n* = 491, 89.6%) used specifically medicinal plants to treat a disease or a health disorder, from which 20.1% (*n* = 110) were habitual (more than 4 times/month) consumers and 69.5% (*n* = 381) were occasional users (1–4 times/month). Only 10.4% of respondents (*n* = 57) had never consumed medicinal plants in the last 12 months (Table 2).

**Table 2:** Frequency of use of herbal products (referring to the last 12 months) with therapeutically purposes among participation sample, according to range of age

Frequency of use of herbal products	Age		
	18–44 n (%)	45–64 n (%)	≥ 65 n (%)
Frequently^a^	48 (8.8%)	39 (7.1%)	23 (4.2%)
Occasionally^b^	256 (46.7%)	102 (18.6%)	23 (4.2%)
Never	35 (6.4%)	13 (2.4%)	9 (1.6%)

^a^ > 4 times/month

^b^1–4 times/month

**Fig. 2:**
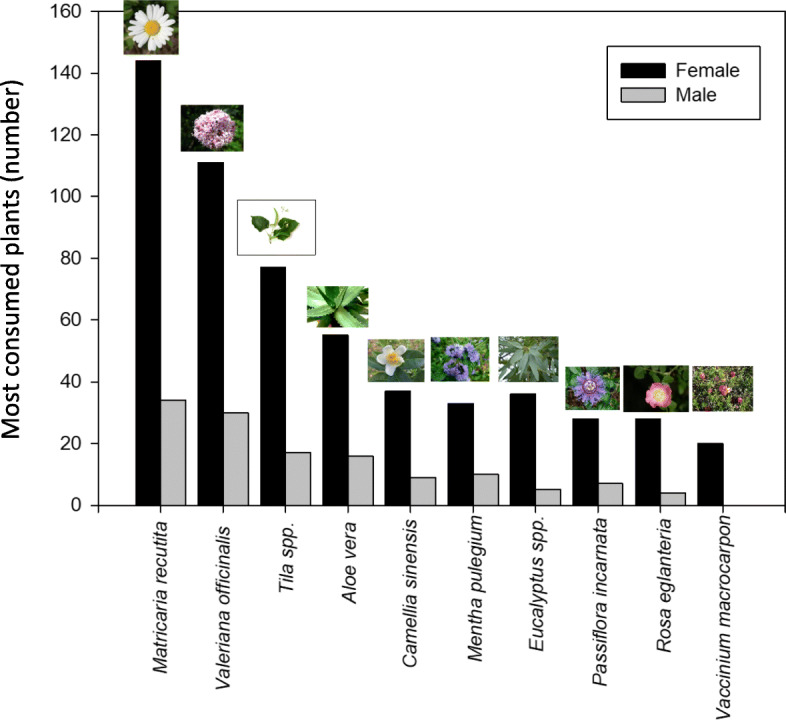
The ten most consumed medicinal plants by the population of the Autonomous Community of Madrid in Spain according to the gender (n total = 543; n female = 382 and n male = 161)

A total of 78 medicinal plants used for health problems, were identified in this study (Table 3). The average consumption was 2.3 medicinal plants by participant. The ten most commonly used medicinal plants were *Matricaria recutita* L. (24.8%), *Valeriana officinalis* L. (20.5%), *Tilia* spp. (13.6%), *Aloe vera* L. (9%)*, Camellia sinensis* (L.) Kuntze (7.1%), *Mentha pulegium* L. (6.9%), *Eucalyptus* spp. (5.8%), *Passiflora incarnata* L. (5.2%), *Rosa eglanteria* L. (4.8%) and *Vaccinium macrocarpon* Ait. (3.7%) (Fig. 2)*.* Some of these plants were also consumed in combined preparations, such as *Valeriana officinalis*, *Passiflora incarnata* and *Eschscholzia californica* Cham; however, these mixtures have not been taken into account in the study.

**Table 3:**
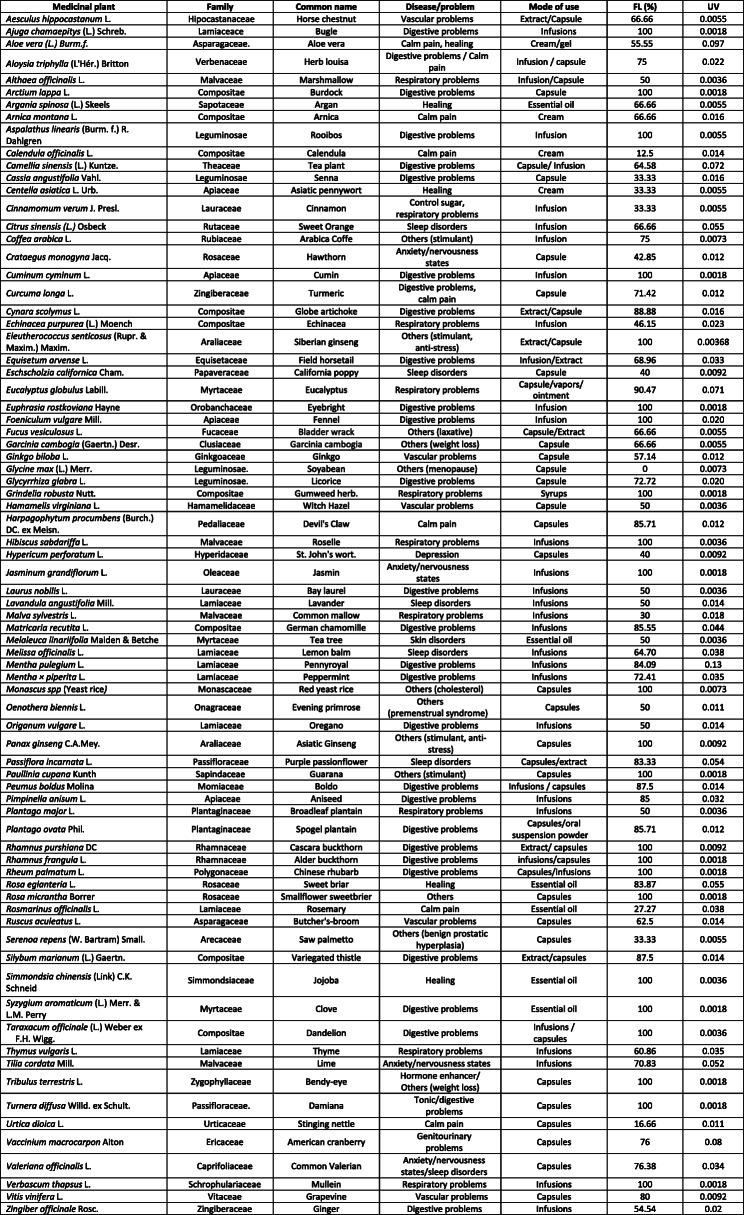
List of reported medicinal plants used (botanical name, family, disease/problem, mode of use, FL and UV)

The uses of the medicinal plants were grouped into 12 categories. The most common therapeutic use (Table 4a) was for digestive problems such as intestinal gas and stomach cramps (*n* = 252, 69%), followed by sleep disorders (*n* = 211, 57.8%), anxiety and nervousness states (*n* = 166, 45.5%) and, respiratory problems such as bronchitis and common cold (*n* = 93, 25.5%). Other less common therapeutically uses were genitourinary problems (*n* = 42, 11.5%), vascular problems (*n* = 31, 8.5%), blood pressure control (*n* = 12, 3.3%), blood sugar levels (*n* = 8, 2.2%) and depression (*n* = 8, 2.2%).

**Table 4:** (A) Main uses for medicinal plants among the survey sample population. (B) Herbal products most used for therapeutic purposes among the population interviewed. Several possible answers were possible for both questions

	**Age**		
	**18–44 n, (%)**	**45–64 n, (%)**	**≥ 65 n, (%)**
**A)**			
**Clinical purposes**			
Anxiety / nervousness states	118 (32.3%)	38 (10.4%)	10 (2.7%)
Blood pressure control	4 (1.1%)	5 (1.4%)	3 (0.8%)
Calm the pain	45 (12.3%)	20 (5.5%)	6 (1.6%)
Control sugar	3 (0.8%)	2 (0.5%)	3 (0.8%)
Depression	4 (1.1%)	2 (0.5%)	2 (0.5%)
Digestive problems	141 (38.6%)	85 (23.3%)	26 (7.1%)
Genitourinary problems	24 (6.6%)	14 (3.8%)	4 (1.1%)
Wound healing	54 (14.8%)	16 (4.4%)	1 (0.3%)
Others	54 (14.8%)	39 (10.7%)	10 (2.7%)
Respiratory problems	55 (15.1%)	34 (9.3%)	4 (1.1%)
Sleep disorders	132 (36.2%)	65 (17.8%)	14 (3.8%)
Vascular problems	12 (3.3%)	17 (4.7%)	2 (0.5%)
**B)**			
**Forms of consumption of medicinal plants**		**Answers n, (%)**	
Creams (i.e. *Aloe vera*, *Rosa eglanteria*, *Arnica montana*)		121 (24.8%)	
Essential oils (i.e. *Eucalyptus spp.*)		84 (17.2%)	
Herbal teas (i.e. *Equisetum arvense*)		369 (75.8%)	
Syrups *(*i.e. *Eleutherococcus senticosus)*		37 (7.6%)	
Tablets, capsules (i.e. *Valeriana officinalis*, *Vaccinium macrocarpon*, *Passiflora incarnata*)		210 (43.1%)	

The most popular form of consumption was as herbal infusion (*n* = 369, 75.8%), followed by tablets/capsules (*n* = 210, 43.1%) and creams (*n* = 121, 24.8%) (Table 4b).

### Quantitative indices

#### The Fidelity Level (FL)

The results of the Fidelity Level for the 10 most cited medicinal plants showed that the highest values were for *Eucalyptus* spp. (FL 90.47%) for respiratory conditions followed by *Matricaria recutita* (85.55%) and *Mentha pulegium* (84.09%) for digestive problems treatment and, *Valeriana officinalis* (76.38%) for insomnia (Table 5).

**Table 5:** Fidelity Level (FL) of the ten most consumed medicinal plants

Medicinal Plants	Main therapeutic uses	No. of claimed uses reports	FL (%)
*Valeriana officinalis* L.	Insomnia	110	76.38
*Aloe vera* L.	Wound healing	40	55.55
*Matricaria recutita* L.	Digestive problems (i.e. flatulence, stomatitis, and gastrointestinal spasms)	154	85.55
*Tilia spp.*	Anxiety	68	70.83
*Rosa eglanteria L.*	Wound healing	26	36.11
*Camellia sinensis* (L.) Kuntze	Asthenia	27	49.09
*Mentha pulegium* L.	Digestive problems (i.e. flatulence, dyspepsia)	37	84.09
*Eucalyptus spp.*	Common cold	38	90.47
*Passiflora incarnata* L.	Anxiety	22	59.45
*Vaccinium macrocarpon* Ait.	Cystitis	19	76

#### The Use Value (UV)

UV calculations revealed that the highest value was found for *Mentha pulegium* (UV 0.130) followed by *Aloe vera* (UV 0.097) and *Vaccinium macrocarpon*. (UV 0.080). These were followed by *Camellia sinensis* (UV 0.072) and *Eucalyptu*s spp. (UV 0.071) (Table 6).

**Table 6:** Use Value (UV) of the ten most consumed medicinal plants

Plant specie	Common name	Part(s) used	Methods of use	Reported uses (per claimed respondents)	UV
*Valeriana officinalis* L.	Valerian	Root	Oral, infusion	Anxiety / nervousness states, blood pressure control, gastrointestinal disorder, sleep disorders	0.034
*Aloe vera* L.	Aloe vera	Gel	Topical	Anxiety / nervousness states, calm pain, gastrointestinal disorders, sleep disorders, vascular problems, wound healing,	0.097
*Matricaria recutita* L.	Chamomile	Flower	Infusion	Anxiety / nervousness states, blood pressure control, calm pain, gastrointestinal disorders, genitourinary problems, sleep disorders, wound healing.	0.044
*Tilia spp,*	Tila	Leaves	Infusion	Anxiety / nervousness states, blood pressure control, calm pain, gastrointestinal disorders, sleep disorders.	0.052
*Rosa eglanteria* L.	Rose Hip	Oil	Topical	Anxiety / nervousness states, sleep disorders, vascular problems wound healing.	0.055
*Camellia sinensis* (L.) Kuntze	Thea	Leaves	Infusion	Anxiety / nervousness states, gastrointestinal disorders, sleep disorders,	0.072
*Mentha pulegium* L.	Pennyroyal	Summit	Infusion	Anxiety / nervousness states, blood pressure control, calm pain, gastrointestinal disorders, sleep disorders.	0.13
*Eucalyptus spp.*	Eucalyptus	Leaves	Topical	Anxiety / nervousness states, gastrointestinal disorders, respiratory disorders,	0.071
*Passiflora incarnata L.*	Passiflora	Aerial part	Infusion	Anxiety / nervousness states, sleep disorders	0.054
*Vaccinium macrocarpon* Ait	Red blueberry	Fruit	Oral	Calm pain, genitourinary problems	0.08

#### Informant Consensus Factor (ICF)

The highest ICF value found corresponds to metabolism and depression (ICF = 1) followed by pain (ICF = 0.97), insomnia (ICF = 0.96) and anxiety (ICF = 0.95) (Table 7).

**Table 7:** Informant Consensus Factor (ICF) per medicinal plant category

Ailment category	Number of claimed medicinal plants	Number of claimed citations	ICF
Anxiety / nervousness states	9	166	0.95
Blood pressure control	3	12	0.81
Calm the pain	3	71	0.97
Control Sugar	1	8	1
Depression	1	8	1
Gastrointestinal disorder	32	252	0.87
Genitourinary problems	5	42	0.90
Respiratory disorders	17	93	0.82
Sleep disorders	8	211	0.96
Vascular problems	5	31	0.86
Wound healing	5	71	0.94

### Place of acquisition preferences and therapeutic resources

Regarding to the place where herbal products were acquired, almost half of the participants preferred pharmacies (*n* = 253, 51.9%) followed by herbal shops (*n* = 209, 42.9%) and supermarkets (*n* = 170, 34.9%), being. The internet resulted in the last position (2.7%) (Table 8).

**Table 8:** People who have recommended the use of medicinal plants, place of acquisition and sources of information among the general population surveyed. Several possible answers were possible for both questions

Questions	Possible responses	n (%)
Who recommends the medicinal plants you use?	Doctor recommendation	67 (13.7)
	Own initiative	216 (44.3)
	Pharmacists advice	162 (33.3)
	Recommended by friends / family / acquaintances	226 (46.4)
Where do you acquire mainly medicinal plants?	Supermarkets	170 (34.9)
	Herbal shops	209 (42.9)
	Internet	13 (2.7)
	Others (i.e. street market)	36 (7.4)
	Pharmacy	253 (51.9)
Where do you mainly get information about the uses of medicinal plants?	Doctor	71 (14.6)
	Family and friends	234 (48.1)
	Internet	160 (32.8)
	Other means of communication (magazines, TV ...)	82 (16.8)
	Pharmacist	210 (43.1)

Most interviewers initiated the consumption of medicinal plants for prevention and treatment following the recommendations of friends and family (*n* = 226, 46.4%), being less who started by their own initiative (*n* = 216, 44.3%) (Table 8). The information concerning the therapeutically uses of medicinal plants came mainly from family and friends (*n* = 234, 48.1%), followed by pharmacist (*n* = 210, 43.1%) and the internet (*n* = 160, 32.8%) (Table 8).

### Subjective perception of risks and precautions of medicinal plants

Half of the respondents (*n* = 227, 46.6%) believed that medicinal plants could cause adverse reactions such as conventional drugs do while the other half of the sample population did not (*n* = 260, 53.4%). Moreover, it was investigated if any of the respondents had suffered any side effect when consuming herbal products for therapeutic purposes. Of those respondents, 17 (3.5%) reported that they had suffered some adverse reaction such as anxiety, tachycardia, dizziness and gastrointestinal symptoms (Table 9).

**Table 9:** Survey responses related to side effects of medicinal plants among the general population surveyed

Questions	Possible responses	n (%)
Do you think that medicinal plants may cause side effects?	Yes	227 (46.6)
	No	260 (53.4)
Have you had any reaction or side effect when consuming medicinal plants?	Yes	17 (3.5)
	No	470 (96.5)
If the previous answer is **YES:**	With which medicinal plant or herbal product? / What side effect or reaction?	
Chamomile / vomiting		
Dandelion / dizziness		
Ginseng / nervous, diarrhea and tachycardia		
Guarana / tachycardia Sen / diarrhea and tachycardia		
St. John’s wort/ interaction similar to the shock		
Tea / anxiety, palpitations		
Valerian / sleepiness the next day		

The potential risk in respect of interactions between medicinal plants and conventional drugs was also investigated. Several respondents have consumed medicinal plants along with conventional medicines (*n* = 103; 21.1%) (Table 10a). Generally, patients do not perceive the need to separate medicinal plants consumption from other drugs. Moreover, interviewees have acknowledged not have received information from health institutions about potential medicinal plants and conventional drugs interactions.

**Table 10:**
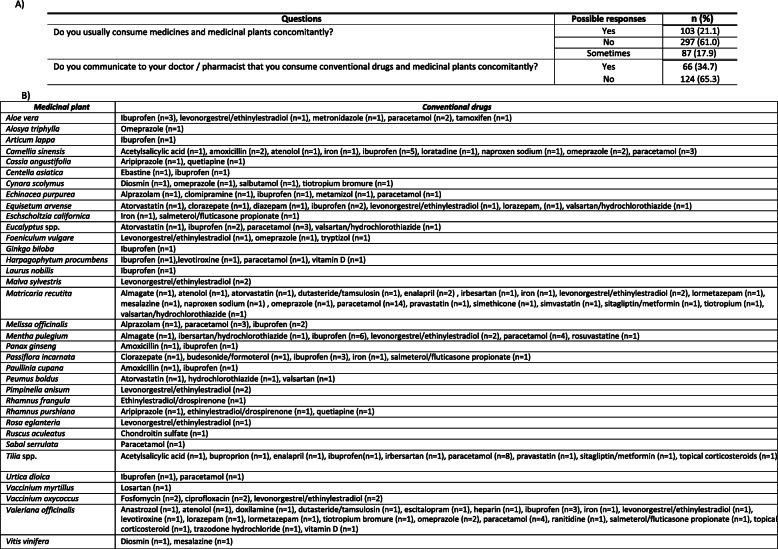
(A) Survey responses related to concomitant consumption of medicinal plants and conventional drugs. (B) Main conventional drugs and medicinal plants that are consumed concomitantly (n = number of there associations have been reported in the survey)

It is revealed that there are several different medicinal plants which were concomitantly consumed with conventional drugs (ibuprofen, levonorgestrel/ethinylestradiol, paracetamol and omeprazole) (Table 10b). It is concluded that *Matricaria recutita* and *Valeriana officinalis* were the medicinal plants most commonly consumed together with conventional drugs.

The percentage of patients who did not inform doctors or pharmacists of medicinal plants consumption while using other medicines was 65.3% (Table 10a).

## Discussion

This work reveals new insights and greater knowledge about the main reasons and current consumption mode of medicinal plants in the population of the Autonomous Community of Madrid for health benefits.

The Community of Madrid has a very varied population and it is very densely populated. Therefore, data from our study were compared with those available from the Institute of Social Sciences to find out whether the surveyed population is representative of the population of this Spanish region. As evidenced demographic parameters are representative (i.e. active population percentage which is 43.6% and range of age which are 55.3% for 18–44, 27.3% for 45–64 and 17.3% for ≥65 years) [16].

Regarding medicinal plants, it was unconcluded that it was higher than the one estimated for other Spanish cities [12]. The main reasons for this finding are the consumer’s perception of efficacy and safety as well as the easy access. In this study, the most common consumption pattern of medicinal plants is young women, between 18 and 44 years of age, with higher education. There is statistically significant differences in consumption frequency related to gender respondents, being higher in women (*P* < 0,001). This high prevalence in the preference of medicinal plants by the female gender has been also confirmed in previous studies [28]. As surveys have been conducted in different health centers, the fact that participants were predominantly women may be due that visits to pharmacies, nurses and doctors in Spain are more frequent in women [29] alongside satisfaction with complementary and alternative medicines [30]. Moreover, a statistically significant finding related to age ranges was found [respondents aged 18–44 consumed medicinal plants more often than those in 45–64 age range (*P* = 0,010) and even more often than those ≥65 years (*P* < 0,001)]. This pattern, contrasts with studies performed in other parts of Europe where the frequency of consumption is higher in older people rather than in younger people [31]. Moreover, studies from the USA found that medicinal plants consumption is more frequent in middle-aged people [10]. These differences may lie in the area where study was conducted, economic level and consumer trends. Particularly, the Autonomous Community of Madrid has the highest Gross Domestic Product per capita in Spain. In addition, it is one of the Spanish regions most influenced by urbanization and where there is not such a strong connection to traditional use of medicinal plants as in other areas of Spain. Furthermore, there is a growing trend, especially amongst younger people with higher educational level, to use natural products to succeed a healthy lifestyle and mentality [3, 31].

One of the limitations found in former published studies on prevalence of medicinal plants consumption, unlikely to the one presented, is on the one hand that “medicinal plants” concept is not properly defined, and on the other hand, a list of medicinal plants is providing limiting the knowledge of their use [5]. Of the 78 identified plants, women reported using 72 while men reported 49. Moreover, most people surveyed use them appropriately in relation to diseases for which they are found to be effective. There were no significant differences (*p* = 0.242) in medicinal plants consumption between female and male. However, preferences for some medicinal plants were found among gender. *Melissa officinalis* L, *Cynara scolymus* L., *Echinacea angustifolia* DC, *Equisetum arvense* L. and *Mentha piperita* L. were preferred by women whereas *Vitis vinifera* L. and *Tribulus terrestris* L. were preferred by men. Moreover, in this study, *Vaccinium macrocarpon* Ait. Consumption was exclusive to women in order to prevent uncomplicated acute lower urinary tract infections recurrence. Women’s urethra is shorter than that of men’s allowing bacteria rapid access to the urinary bladder [32].

It is necessary to emphasize that some of the medicinal plants consumed by the population of the Autonomous Community of Madrid are considered as threatened/vulnerable/endangered by the IUCN Red List. These plant species include in this Red list are *Aesculus hippocastanum* (vulnerable), *Arnica montana* (least concern), *Coffea arabica* (endangered), *Ginkgo biloba* (endangered), *Laurus nobilis* (least concern), *Rhamnus purshiana* (least concern) and *Tilia cordata* (least concern). Particularly, those plant species classified as least concern are not considered to be at threat from extinction and, the future conservation actions are aimed at controlling agriculture practices and include an international legislation. However, *Aesculus hippocastanum* is classified as vulnerable because this plant species suffer from severe defoliation by the invasive insect pest *Cameraria ohridella.* The conservation actions consists on *Cameraria ohridella* control and research, ex situ cultivation and to reduce human impacts. On the other hand, *Coffea arabica* and *Ginkgo biloba* are endangered plant species. The main threats to *Coffea arabica* are pests (i.e. *Hypothenemus hampei*), diseases (i.e. Coffee Berry Disease), deforestation (mainly in Ethiopia) and climate change (i.e. high temperatures). There are several conservation actions for *Coffea arabica* such as ex-situ conservation and, education and awareness programs. Finally, *Ginkgo biloba* is threatened because its logging and wood harvesting. The conservation action for this specie has been widespread in cultivation. It is therefore important that investigations with these species follow the guidelines “IUCN Policy Statement on Research Involving Species at Risk of Extinction” that guarantee the increase and survival of these plant species, bearing in mind that the conservation of these research sources is of clear scientific interest, and in the case of our study, of great therapeutic interest [33–39].

Regarding forms of consumption, the effectiveness of medicinal plants depends on the correct use and preparation. Decoction and infusion are the main preparation methods for herbal teas of roots, barks and seeds. Herbal teas are closely linked to self-medication, being this form of administration not suitable for active principles with narrow therapeutic margin. Tablets/capsules are commonly used for medicinal plants oral administration because of good bioavailability, therapeutic adherence and patient comfort [40].

Concerning accessibility to medicinal plants, most of the herbs are freely available in different places for its acquisition, even at supermarkets (i.e. *Matricaria recutita*, *Camellia sinensis* and *Mentha pulegium*) whereas there are other medicinal plants that are only available in local pharmacies and herbal shops (i.e. *Verbascum thapsus* and *Ajuga chamaepitys*). Participants’ perception is that medicinal plants dispensed in pharmacies have better quality and efficiency than those from other acquisition places; however, medicinal plants bought in pharmacies are more expensive than in other sales establishments. This explains why the purchase of medicinal plants in supermarkets and herbal shops is very high. This pattern of herbal products acquisition for therapeutic purposes has also been observed in other countries [41]. However, within Spain, patients from a social security primary health care center in Barcelona bought medicinal plants first in herbal shops, then in supermarket and in pharmacies in third place [12]. The role of the pharmacist is consolidated as the health professional and expert in medicinal plants and pharmacy offices as a reference in the dispensation of medicinal plants, offering quality guarantees.

Due to the wide traditional utilization of medicinal plants and the limited existing clinical trials, there is a lack of scientific evidence on the efficacy and safety of medicinal plants [4]. Adverse drug reactions is defined as “all noxious and unintended responses to a medicinal product” [42, 43]. There is a common perception of safety of medicinal plants as “natural” and “harmless”, which could lead to an under-reporting of adverse reactions. Adverse reactions may be due both to medicinal plants and to other factors (i.e. adulteration, lack of botanical identification) [44]. Studies conducted on natural products’ perception for health, show an increase in the demand for information about medicinal plants [45, 46]. It is necessary to include medicinal plants consumption in the usual medical history to identify possible adverse reactions and drug interactions [47]. Many health professionals have not received academic preparation on medicinal plants during their Degree studies [48]. In Spain, only pharmacists receive university education on medicinal plants. This lack of knowledge is a limiting factor when health professionals recommend medicinal plants and identify possible adverse reactions and interactions. The need to include medicinal plants in undergraduate training to the rest of health professionals is presumed.

Currently, there are a paucity of robust data on interactions between medicinal plants and conventional medicines [49]. However, it has been found that certain plants can lead to therapeutic inefficiency or drug toxicity. There is evidence of interactions for *Hyperycum perforatum* L. with digoxin, indinavir and cyclosporines [50]. Moreover, *Ginkgo biloba* L. Mant. Pl. can increase insulin elimination or interfere with omeprazole [51]. Furthermore, and in relation to the medicinal plants, that are more consumed concomitantly in this study, there are evidences of pharmacodynamics interactions between *M. recutita* and lormetazepam, *M. officinalis* and alprazolam, and *V. officinalis* and lormetazepam, increasing hypnotic effect of these benzodiazepines [51]. The clinical effects of the interactions depend on patient (age, genetic and pathologies), medicinal plants (species, dose and duration) and concomitant medication (dose, activity and posology) making it difficult to detect interactions if health personnel do not know its use.

Finally, several participants told that neither they reported medicinal plants consumption to these health professionals nor did they ask. This leads to a potential underreporting of adverse reactions and interactions with medicinal plants and, supports the need in the academic training of health sciences personnel to include subjects of medicinal plants in undergraduate degree.

## Conclusions

In this paper, we have explored medicinal plant uses, consumption patterns and attitude towards medicinal plants of the population of the Autonomous Community of Madrid that attend health-related centers. This study shows that although the Autonomous Community of Madrid is not a region of Spain with a long tradition in the use of medicinal plants, many inhabitants currently use herbal products (i.e. *M. recutita*, *V. officinalis*, *Tilia*, *A. vera*. and *C. sinensis*) to treat, mainly, minor health problems (i.e. digestive problems and sleep disorders). All the reported medicinal plants have been extensively used in different countries, not identifying neither new records nor new therapeutic activities. These medicinal plants are mainly acquired in pharmacies, herbal shops and supermarkets. The most common consumer pattern of medicinal plants are young women between 18 and 44 years of age with higher education. It has been proved that one of the main reasons for the use of medicinal plants is that the surveyed population has the perception that being natural means harmless.

Moreover, in the present work a correct use of medicinal plants-therapeutic benefits has been detected. However, the high percentage of self-medication may increase the problem of lack of adverse reaction registration and/or drug interactions. Medicinal plants consumption is a matter to consider in the control of pharmacological treatments of the patients. This will guarantee safety, efficacy and quality in the use of medicinal plants, thus constituting an integral health system. According to the results of the study, the need for studies and research to predict the future use of medicinal plants is verified to ensuring the best quality of traditional herbal remedy.

Furthermore, taking into account that studies on current uses of medicinal plants in Spain are very limited, it would be interesting in future research to approach other regions in Spain to have deeper knowledge of the current situation, using and adapting the tolls of this work.

## Supplementary information

**Additional file 1.** Survey on medicinal plants. The questionnaire was developed in Spanish language and designed for this study.

## Data Availability

The data will be accessible by contacting the corresponding author of this study.

## Abbreviations

CAM: Complementary and alternative medicine
FL: Fidelity Level
UV: Use Value
ICF: Informants Consensus Factor
