# Supplementary material for: Current uses and knowledge of medicinal plants in the Autonomous Community of Madrid (Spain): a descriptive cross-sectional study
Source: BMC Complement Med Ther. 2020 Oct 14;20:306. doi: 10.1186/s12906-020-03089-x (PMC7557077; doi:10.1186/s12906-020-03089-x)
Supplement: Supplementary file 1 — Additional file 1. Survey on medicinal plants. The questionnaire was developed in Spanish language and designed for this study. [file 12906_2020_3089_MOESM1_ESM.pdf]

# CONSUMPTION AND KNOWLEDGE SURVEY OF MEDICINAL PLANTS AND RELATED PRODUCTS IN THE AUTONOMOUS COMMUNITY OF MADRID

1. **Gender:** Female ☐ Male ☐

2. **Age**: 18-44 ☐ 45-64 ☐ ≥ 65 ☐

3. **Educational level :** Basic education ☐ Vocational training ☐ Higher education ☐

4. **Place of residence:** Postal code ☐

5. **Occupation:** Student ☐ Employers ☐ Unemployed ☐ Pensioners ☐ No answer ☐

Medicinal plants definition: *"Plants that contain properties or compounds that can be used for therapeutic purposes or those that synthetize metabolites to produce useful drugs"*

6. **Frequency with which you consume medicinal plants (referring to the last 12 months):**
   Frequently* ☐ Occasionally** ☐ Never ☐
   ** > 4 times/month ** 1-4 times/month*

7. **What do you use medicinal plants for therapeutic purposes?** (more than one possible option)

   - O Respiratory problems
   - O Digestive problems
   - O Sleep disorders
   - O Anxiety and nervousness states
   - O Calmar dolor
   - O Blood pressure control
   - O Depression
   - O Vascular problems
   - O Genitourinary problems
   - O Sugar levels
   - O Healing
   - O Others: ☐

8. **What medicinal plants have you used?**

☐

9. **Forms in which you have consumed medicinal plants:**
   - O Herbal teas
   - O Tablets/capsules
   - O Essential oils
   - O Creams
   - O Syrups

**10. Who recommends the medicinal plants you use?:**

- O Doctor recommendation
- O Pharmacist advice
- O Friends and family recommendations
- O Own initiative

**11. Where do you acquire mainly medicinal plants?:**

- O Pharmacy
- O Herbal shops
- O Internet
- O Supermarkets
- O Others (i.e. Street markets)

**12.Where do you mainly get information about the uses of medicinal plants?:**

- O Doctor
- O Pharmacist
- O Family and friends
- O Internet
- O Other means of communications (i.e. magazines, TV)

**13. Do you think that medicinal plants may cause side effects / adverse reactions?:**

YES ☐ NO ☐

**14. Have you had any side effects / adverse reactions when consuming medicinal plants?** YES ☐ NO ☐

**15. If the previous answer is YES:**

- **What side effects / adverse reactions?** ☐
- **With which medicinal plant?** ☐

**16. Do you usually consume medicines and medicinal plants concomitantly?** YES ☐ NO ☐ SOMETIMES ☐

**17.If the previous answer is "YES" or "SOMETIMES", please, indicate the name of the medicine and the medicinal plants that you consume concomitantly:**

| Name of the medicine | Name of the medicinal plant |
|---|---|
| | |

**18.If the answer to question number 17 is "YES" or "SOMETIMES":**

**- Do you tell your doctor / pharmacist that you are consuming concomitantly medicines and medicinal plants?**

YES ☐ NO ☐
